# Combining robot-assisted therapy with virtual reality or using it alone? A systematic review on health-related quality of life in neurological patients

**DOI:** 10.1186/s12955-023-02097-y

**Published:** 2023-02-21

**Authors:** Francesco Zanatta, Naima Z. Farhane-Medina, Roberta Adorni, Patrizia Steca, Anna Giardini, Marco D’Addario, Antonia Pierobon

**Affiliations:** 1 Department of Psychology, University of Milano-Bicocca, Milan, Italy; 2 Maimonides Biomedical Research Institute of Córdoba (IMIBIC), Córdoba, Spain; 3 Department of Psychology, University of Córdoba, Córdoba, Spain; 4 Information Technology Department, Istituti Clinici Scientifici Maugeri IRCCS, Pavia, Italy; 5 Psychology Unit of Montescano Institute, Istituti Clinici Scientifici Maugeri IRCCS, Montescano, Italy

**Keywords:** Health-related quality of life, Neurorehabilitation, Robot-assisted therapy, Virtual reality, Systematic review

## Abstract

**Background:**

In the field of neurorehabilitation, robot-assisted therapy (RAT) and virtual reality (VR) have so far shown promising evidence on multiple motor and functional outcomes. The related effectiveness on patients’ health-related quality of life (HRQoL) has been investigated across neurological populations but still remains unclear. The present study aimed to systematically review the studies investigating the effects of RAT alone and with VR on HRQoL in patients with different neurological diseases.

**Methods:**

A systematic review of the studies evaluating the impact of RAT alone and combined with VR on HRQoL in patients affected by neurological diseases (i.e., stroke, multiple sclerosis, spinal cord injury, Parkinson’s Disease) was conducted according to PRISMA guidelines. Electronic searches of PubMed, Web of Science, Cochrane Library, CINAHL, Embase, and PsychINFO (2000–2022) were performed. Risk of bias was evaluated through the National Institute of Health Quality Assessment Tool. Descriptive data regarding the study design, participants, intervention, rehabilitation outcomes, robotic device typology, HRQoL measures, non-motor factors concurrently investigated, and main results were extracted and meta-synthetized.

**Results:**

The searches identified 3025 studies, of which 70 met the inclusion criteria. An overall heterogeneous configuration was found regarding the study design adopted, intervention procedures and technological devices implemented, rehabilitation outcomes (i.e., related to both upper and lower limb impairment), HRQoL measures administered, and main evidence. Most of the studies reported significant effects of both RAT and RAT plus VR on patients HRQoL, whether they adopted generic or disease-specific HRQoL measures. Significant post-intervention within-group changes were mainly found across neurological populations, while fewer studies reported significant between-group comparisons, and then, mostly in patients with stroke. Longitudinal investigations were also observed (up to 36 months), but significant longitudinal effects were exclusively found in patients with stroke or multiple sclerosis. Finally, concurrent evaluations on non-motor outcomes beside HRQoL included cognitive (i.e., memory, attention, executive functions) and psychological (i.e., mood, satisfaction with the treatment, device usability, fear of falling, motivation, self-efficacy, coping, and well-being) variables.

**Conclusions:**

Despite the heterogeneity observed among the studies included, promising evidence was found on the effectiveness of RAT and RAT plus VR on HRQoL. However, further targeted short- and long-term investigations, are strongly recommended for specific HRQoL subcomponents and neurological populations, through the adoption of defined intervention procedures and disease-specific assessment methodology.

**Supplementary Information:**

The online version contains supplementary material available at 10.1186/s12955-023-02097-y.

## Background

Neurological diseases result in a broad spectrum of motor, functional, and cognitive impairments. Consequently, they represent the leading cause of disability and the second leading cause of death worldwide [1]. Contextually to populations growing and ageing, the burden of neurological disorders continues to increase globally. Therefore, urgent solutions from interdisciplinary rehabilitation, innovative approaches and technology-enabled smart healthcare strategies are needed [2]. Notably, in recent decades, the field of neurorehabilitation has shown a growing interest in high technologies, like robotics and virtual reality (VR), due to their potential and multipurpose application to patients’ recovery pathways [3, 4].

Robot-assisted therapy (RAT), for example, has been extensively used in accordance with the principles of motor relearning and neuroplasticity with the purpose of better maximizing afferent input from peripheral joints and providing task-specific stimulation to the central nervous system [3]. In targeting both upper and lower limb impairment, RAT has so far shown several advantages, including the implementation of repetitive, intensive, and task-oriented rehabilitation exercises through a smaller workforce, optimized and customized treatment, and real-time quantitative assessment and monitoring of motor disability [5]. For this purpose, different robotic devices have been used so far (i.e., exoskeletons, end-effectors, soft-robots) [6], reporting promising evidence on diverse rehabilitation outcomes related to both upper (e.g., arm range of motion, hand grip and strength, dexterity) and lower (e.g., gait, balance, mobility) limb impairment. Notably, prior systematic reviews and meta-analyses have reported informative effects in different acute and chronic conditions, including stroke [7, 8], traumatic brain injury (TBI) [9], spinal cord injury (SCI) [10], multiple sclerosis (MS) [11], and Parkinson’s Disease (PD) [12], corroborating the added value of integrating robotic technologies in standard rehabilitation programs across clinical populations.

Although RAT has been shown to enable effective training, deeper investigation to shed light on its broader potential is still needed. For instance, further studies have highlighted the effects of robotic devices when combined with additional technological systems, like VR [13]. VR is defined as a system based on computer-simulated 3D environments (i.e., virtual environments—VEs) that allow the user to interact with virtual objects by the integration of visual, auditory, and haptic feedback [14]. It is acknowledged that three main factors characterize VR, namely immersion, sense of presence, and interactivity. Immersion refers to the degree to which the VE can provide multisensory stimuli originating from a high degree of matching between the cues generated by the systems and the user's actions. Therefore, immersion in and interactivity with VEs affects users' perception, ultimately determining their sense of presence. VR systems can be distinguished into fully-immersive, semi-immersive, and non-immersive devices, depending on the extent to which the interaction with the VE blocks out user’s real-world perception. Examples of fully-immersive VR devices are tools like head-mounted displays (HMD) and cave automatic virtual environment (CAVE). Among the semi-immersive devices, large monitors or projectors provide the users moderate immersion, while non-immersive tools include simpler devices such as PCs or tablets. Through the implementation of diverse VEs at different levels of immersion VR has so far offered multimodal stimulation and multisensory feedback during training. This has not only provided patients with more engaging therapy sessions, contributing to enduring practice and strengthening performance awareness, but it has also enabled deeper stimulation, ultimately inducing cortical and subcortical synaptic-level changes that are essential for motor relearning [15].

Nevertheless, it must be acknowledged that, although robotic and VR devices have so far shared high technological impact, they profoundly differ from each other when considering technical and interactivity issues. Therefore, the applicability of these technological devices and their efficacy still need to be better understood, especially when they are implemented independently or in combination and when they are used with specific neurological populations.

Overall, it must be noted that the introduction of RAT alone or in combination with VR has mainly provided more robust conclusions when taking into account outcomes like motor improvement and functional status change [5, 16]. However, the broader effectiveness of this innovative recovery procedure, including its long-term impact, still needs to be more deeply investigated and confirmed. Health-related quality of life (HRQoL) has so far been considered as an indicator of therapy effectiveness, since it facilitates observing patients’ perceived improvement in terms of their physical and mental state, especially when taking into consideration patients’ real-life environments and health domains. Nevertheless, prior studies have predominantly explored HRQoL as an additional outcome, with the main objective to extend the investigation of RAT efficacy to the secondary effects resulting from motor improvement. Similarly, concurrent investigations have been carried out to include cognitive and psychosocial outcomes. For example, alongside motor recovery, recent studies have highlighted positive changes on depression and anxiety symptoms, increased perceived well-being, improved coping strategies, and enhanced executive function performance [13, 17–19].

Following this line, the adoption of a multidimensional approach in the field of neurorehabilitation, where multiple patient’s health-related domains and their complementarity are considered, still remains an open challenge [20]. Accordingly, it is of paramount concern to provide clearer and wider overview of RAT effects, in particular targeting a wide range of neurological diseases and disorders and by giving more centrality to patient’s non-motor characteristics is of paramount concern. This would not only help future clinical trials to better orient their investigation on disease-specific outcomes, but it would also better address the effects of RAT beyond motor improvement.

For this purpose, a systematic review was conducted on studies investigating the impact of RAT, alone or combined with VR, on HRQoL in patients affected by neurological diseases. Specifically, the present study aimed at describing the evidence on short- and long-term post-intervention changes regarding HRQoL, including how this outcome was evaluated across neurological populations. In addition, a secondary systematic synthesis was conducted on the non-motor outcomes (e.g., cognitive, psychological) that were investigated concurrently.

## Methods

A preliminary check on registered or ongoing similar systematic reviews was conducted on the International Prospective Register of Systematic Review (PROSPERO) platform. No results were provided and, thus, the systematic review protocol was registered (ref. CRD42022367228).

The current study belongs to a broader project called PHTinRehab Study (Perception of High Technology in Rehabilitation: a prospective real-life Study on usability, effectiveness, and health-related quality of life), approved by the Ethics Committee of the Clinical Scientific Institutes Maugeri IRCCS (February 2021, protocol n. 2517CE).

### Search strategy and studies selection

The preferred Reporting Items for Systematic Reviews and Meta-Analyses (PRISMA) guidelines were followed [21]. Preliminary electronic searches of PubMed, Web of Science, Cochrane Library, CINAHL, Embase, and PsychINFO were performed on 11th January 2022 by applying the following search query for all databases: (rehabilitation) AND ((robot*) OR (virtual reality)) AND (quality of life). Even though the present study aimed to retrieve records specifically focused on neuromotor rehabilitation, the general term ‘rehabilitation’ was preferred to ensure full retrieval despite its applicability to different clinical fields (e.g., cardiac-pulmonary rehabilitation, cognitive rehabilitation). Therefore, the identification of the eligible studies was checked throughout the records screening procedure. Furthermore, to optimize studies identification according to the eligibility criteria (Table 1), additional mutual filters (i.e., year of publication timespan, article language) were defined consistently for each electronic database. During the systematic review process, a reference management and bibliography-creating software (EndNote Web) was implemented.

**Table 1:** Eligibility criteria

*Inclusion criteria*
HRQoL evaluation following RAT or RAT plus VR-based neurorehabilitation
Adult patients affected by neurological diseases
Quantitative and qualitative studies
Original research published in English in a peer-reviewed journal
Year of publication timespan: 2000–2021
*Exclusion criteria*
Other fields of rehabilitation (e.g., VR-based cognitive rehabilitation)
Healthy participants or patients affected by psychiatric disorders
Conference papers, proceedings, study protocols, reviews, commentaries, editorials, position papers

### Risk of bias assessment

The National Institutes of Health (NIH) Quality Assessment Tool for Controlled Intervention Studies and for the Before–After (Pre–Post) Studies With No Control Group were used to evaluate the methodological quality of the records included [22]. The first one comprised 14 items assessing the quality of randomization, treatment allocation, blinding, similarity of study groups at baseline, dropout management, adherence to treatment, outcome measures validity, power calculation, hypothesis testing, and intention-to-treat analysis. The second checklist comprised 12 items evaluating the quality of the study question, eligibility criteria, sample representativeness, intervention description, outcome measures validity, blinding, follow-up rate, and statistical analyses. For both checklists, each item was rated as yes, no, or not reported, assigning one point for every ‘yes’ answer. Based on this evaluation, studies were classified according to quality rating: ‘Poor’, ‘Fair’, or ‘Good’ (details on the NIH Quality Assessment questions and the scoring procedures can be found in the supplementary material). The studies were evaluated by three researchers (F.Z., N.F., R.A.) working independently. Any discrepancies were discussed until a consensus was reached. The studies evaluated as having a higher risk of bias were not excluded, but their quality was considered in the interpretation of the results, as appropriate [23].

### Data extraction and synthesis

Following eligibility criteria discussion and agreement of all authors, progressive exclusion of the non-eligible records was performed starting from the title, then the abstract, and finally the full-text. Two authors (F.Z. and N.F.) completed the entire review process working independently. Disagreements were solved through periodically planned discussions involving all authors. Each identified article was then screened multiple times until sufficient understanding of the study aim, design, and outcomes was obtained. Of the included studies, a wide range of data was then extracted and reported in a structured table. Due to its extent, it was provided as supplementary material. The table includes: author(s), year of publication, nation where the study was conducted, the nation’s Human Development Index (HDI) [24] and the related rank, professional specialty field of the research group, study design (including if studies were multicentric, pilot, funded, if they included any follow-up evaluations and related duration), patients’ characteristics (i.e., disease, type of hospitalization, sample size, mean age, ethnicity, any prior experience using technological devices), study purpose, rehabilitation outcomes, commercial name of technological devices, robotic device typology (i.e., exoskeleton, end-effector, soft-robot), level of VR immersion (i.e., non-immersive, semi-immersive, fully-immersive), intervention characteristics (i.e., duration in weeks, total number of sessions, session duration in minutes), HRQoL, psychological variables, motor and functional outcome measures used, and main results. Descriptive statistics were calculated on the main characteristics of the studies, whereas the main results were meta-synthetized through narrative analysis. No specific statistical software or tool was implemented for data synthesis.

## Results

### Flow of studies through the review

**Fig. 1:**
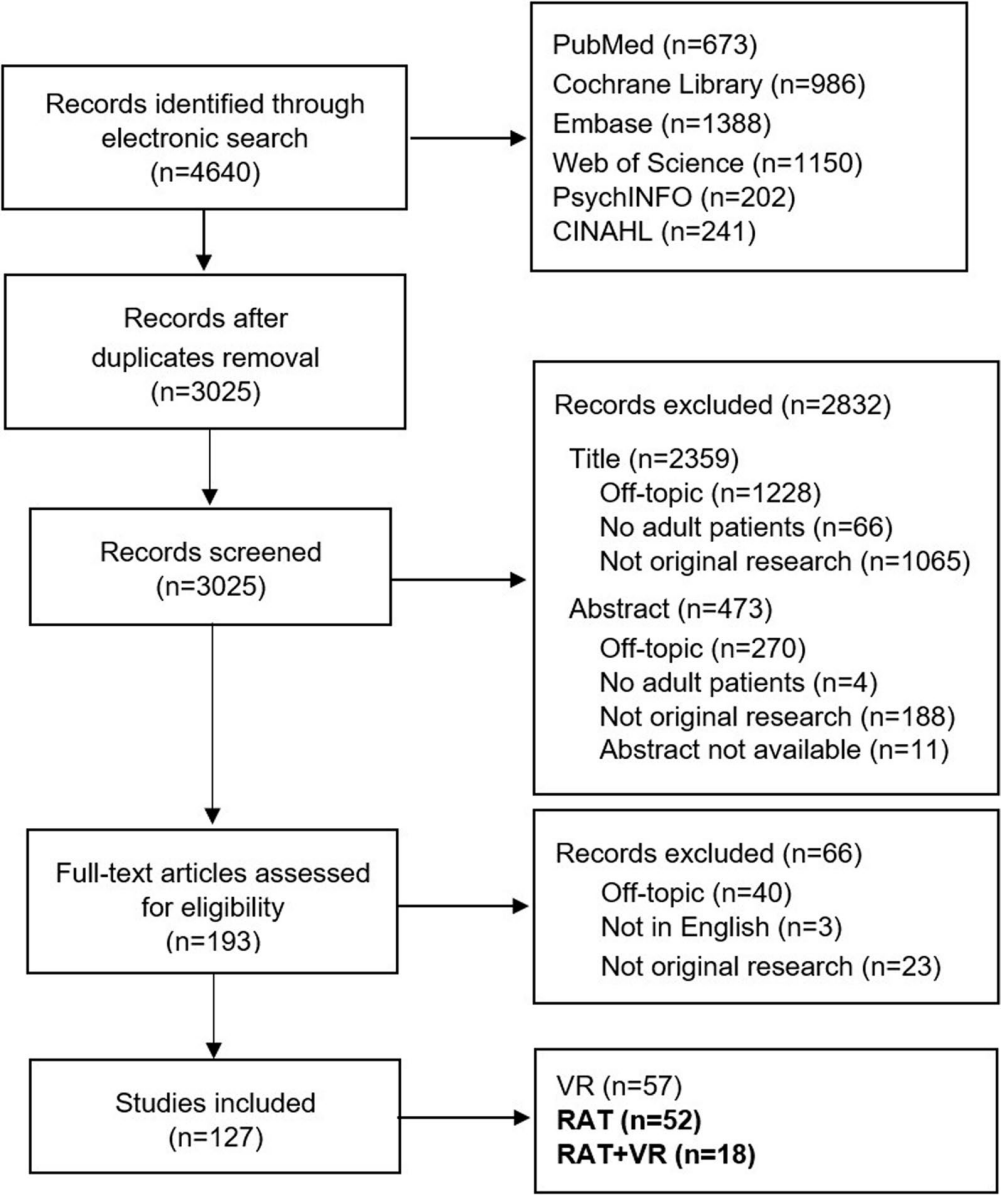
Flow chart of the review process

From the initial electronic search, a total of 4640 records were retrieved. After duplicates were identified and removed, 3025 studies underwent the screening procedure. A total of 2832 records were excluded by title and abstract screening. The remaining 193 were screened by full-text. Of these, 127 met all the inclusion criteria. Additional records were not identified by further hand searching. Figure 1 shows the flow diagram of the studies selected, including the reasons for exclusion. Most of the excluded records were considered as off-topic (n = 1538). These were not strictly focused on RAT, but belonged to different fields of rehabilitation (e.g., traditional treatment exclusively, cognitive rehabilitation). Others specifically involved non-adult patients (n = 70), whereas other records (n = 1276) were identified as non-original research (e.g., study protocols, proceedings). Of the included studies, a total of 57 reported results on the effects of VR-based rehabilitation exclusively. The remaining provided evidence on RAT alone (n = 52) or in combination with VR (n = 18). The present work specifically describes the results from the implementation of RAT alone or plus VR. Thus, the final number of studies included in this systematic review is 70. The results on the effects of VR will be presented in a separate work.

### Risk of bias

Of the studies evaluated with the Controlled Intervention Studies checklist (n = 48), most (n = 28, 58.3%) were classified as having ‘fair’ methodological quality. 33.4% of the studies met most of the criteria and were evaluated as ‘good’ (n = 16), whereas only 4 studies (8.3%) were labelled as ‘poor’. The remaining 22 studies were assessed with the Before-After (Pre-Post) Studies With No Control Group scale. The majority of these (n = 16, 72.7%) had ‘fair’ methodological quality, while four (18.2%) were classified as ‘good’ and the final two (9.1%) as ‘poor’ quality.

Overall, only 11.4% of the included studies (n = 70), were therefore evaluated as having a potentially high risk of bias. Details on the evaluation for each study are reported as supplementary material.

### Characteristics of the included studies

A full report of the data extracted from the included studies is presented in a structured synoptic table (Additional file 1: Append ix 1) [13, 25–93].

### Design

The main characteristics regarding the design of the included studies are summarised in Tables 2 and 3. Most (65.7%) were published in the last five years and were conducted in European countries (55.7%). Data extracted on the research groups’ specialty fields denote a multidisciplinary contribution to the investigation on the current topic, with the majority of the research groups having their main expertise in rehabilitation medicine (92.6%), neurology (41.4%), engineering (25.7%), neuroscience (14.3%), and psychology (12.9%). Most of the studies (62.9%) were randomised controlled trials (RCTs), followed by pre-post clinical trials without a control group (31.4%) and quasi-experimental studies (5.7%). More than half of the studies included follow-up evaluations (55.7%), were single-centered (75.7%), and were funded (61.4%). In conclusion, almost one study out of three was a pilot (28.6%).

**Table 2:** Main characteristics of the included studies (n = 70)

Year of publication	n (%)	Nation^a^	n (%^b^)	Research group specialty field	n (%^b^)
2016–2021	46 (65.7)	Europe	39 (55.7)	Rehabilitation Medicine	65 (92.6)
2011–2015	18 (25.7)	America	21 (30.0)	Neurology	29 (41.4)
2000–2010	6 (8.6)	Asia	17 (24.3)	Engineering^c^	18 (25.7)
		Oceania	2 (2.9)	Neuroscience	10 (14.3)
				Occupational Physiatry	9 (12.9)
				Psychology	9 (12.9)
				Physiology	6 (8.6)
				Geriatrics and orthopedics	5 (7.1)
				Public Health	3 (4.3)

^a^Europe (i.e., Denmark, Germany, Italy, Netherlands, Norway, Romania, Spain, Sweden, Switzerland, Turkey, United Kingdom), America (i.e., Canada, United States of America), Asia (i.e., China, India, Israel, Japan, South Korea, Taiwan), Oceania (Australia)

^b^Non-cumulative percentages

^c^Biomedical, Mechanical, Computer Engineering

**Table 3:** Study design characteristics of the included studies (n = 70)

Study design	n (%)	Follow-up	n (%)	Funding	n (%)	Multicentric	n (%)	Pilot Study	n (%)
RCTs	44 (62.9)	Yes^a^	39 (55.7)	Yes	43 (61.4)	Yes	17 (24.3)	Yes	20 (28.6)
Pre-post Clinical Trial (no control group)	22 (31.4)	No	31 (42.3)	No	27 (38.6)	No	53 (75.7)	No	50 (71.4)
Non-RCT	4 (5.7)								

^a^Follow-up range: 1–36 months

### Participants

Table 4 shows the main characteristics of participants. The sample size of the included studies ranged from 3 to 224 patients (total of all studies, n = 2956). The 37.1% of the studies included fewer than 30 participants and, according to risk of bias assessment, only 17.2% reported that sample size was sufficiently large to detect results with appropriate statistical power. Most of the participants included were outpatients (44.3%), suffered from stroke (54.3%), MS (21.4%), SCI (15.7%), or PD (15.7%). Finally, only four studies reported participants’ ethnicity [54, 58–60].

**Table 4:** Participants’ characteristics in the included studies (n = 70)

Patients	n (%)	Disease	n (%)
Outpatients	31 (44.3)	Stroke	38 (54.3)
Inpatients	15 (21.4)	Multiple Sclerosis	15 (21.4)
Both	3 (4.3)	Spinal Cord Injury	11 (15.7)
Not defined	21 (30.0)	Parkinson’s Disease	3 (4.3)
		Other^a^	3 (4.3)

^a^Hereditary Spastic Paraplegia, Neuromuscular diseases, Orthopedics

### Intervention

Robotic and VR devices typology, including the main characteristics of the interventions are summarized in Table 5. Overall, 74.3% of the studies implemented RAT exclusively, while the remaining 25.7% coupled RAT with VR systems. Of the latter, all used non-immersive VR devices, except for one study that also implemented semi-immersive VE [61]. In particular, the studies on RAT mainly implemented exoskeletal devices (59.6%), and particularly targeted rehabilitation outcomes related to lower limb functioning (71.2%), such as gait, balance, mobility, and muscle strength. The studies investigating the effects of RAT plus VR implemented exoskeletal or end-effector robotic devices (44.4%), and mainly provided rehabilitation exercises to target upper limb outcomes (72.2%), including manual dexterity, reaching and grasping abilities, shoulder pain, and spasticity. Regarding intervention duration, RAT alone lasted longer on average when compared to RAT plus VR in terms of overall therapy duration (in weeks), total number of sessions, and session duration (in minutes).

**Table 5:** Main characteristics of the technological devices and of the intervention in RAT (n = 52) and RAT plus VR (n = 18) studies

	RAT	RAT plus VR	Overall
Robot typology, n (%^a^)			
Exoskeleton	31 (59.6)	8 (44.4)	39 (55.7)
End-effector	21 (40.4)	8 (44.4)	29 (41.4)
Soft-robotics	–	3 (16.6)	3 (4.3)
Targeted extremities, n (%)			
Upper Limbs	15 (28.8)	13 (72.2)	28 (40.0)
Lower Limbs	37 (71.2)	4 (22.2)	41 (58.6)
Both	–	1 (5.6)	1 (1.4)
Intervention, mean ± SD (range)			
Overall duration (weeks)	6.5 ± 3.7 (2–24)	4.8 ± 1.5 (3–8)	6.0 ± 3.3 (2–24)
n. of sessions	22.0 ± 10.2 (6–60)	19.6 ± 8.9 (8–40)	21.4 ± 9.9 (6–60)
Session duration (min)	60.1 ± 30.9 (20–180)	55.7 ± 39.7 (30–180)	58.9 ± 33.4 (20–180)

^a^Non-cumulative percentage

### HRQoL outcome measures

**Fig. 2:**
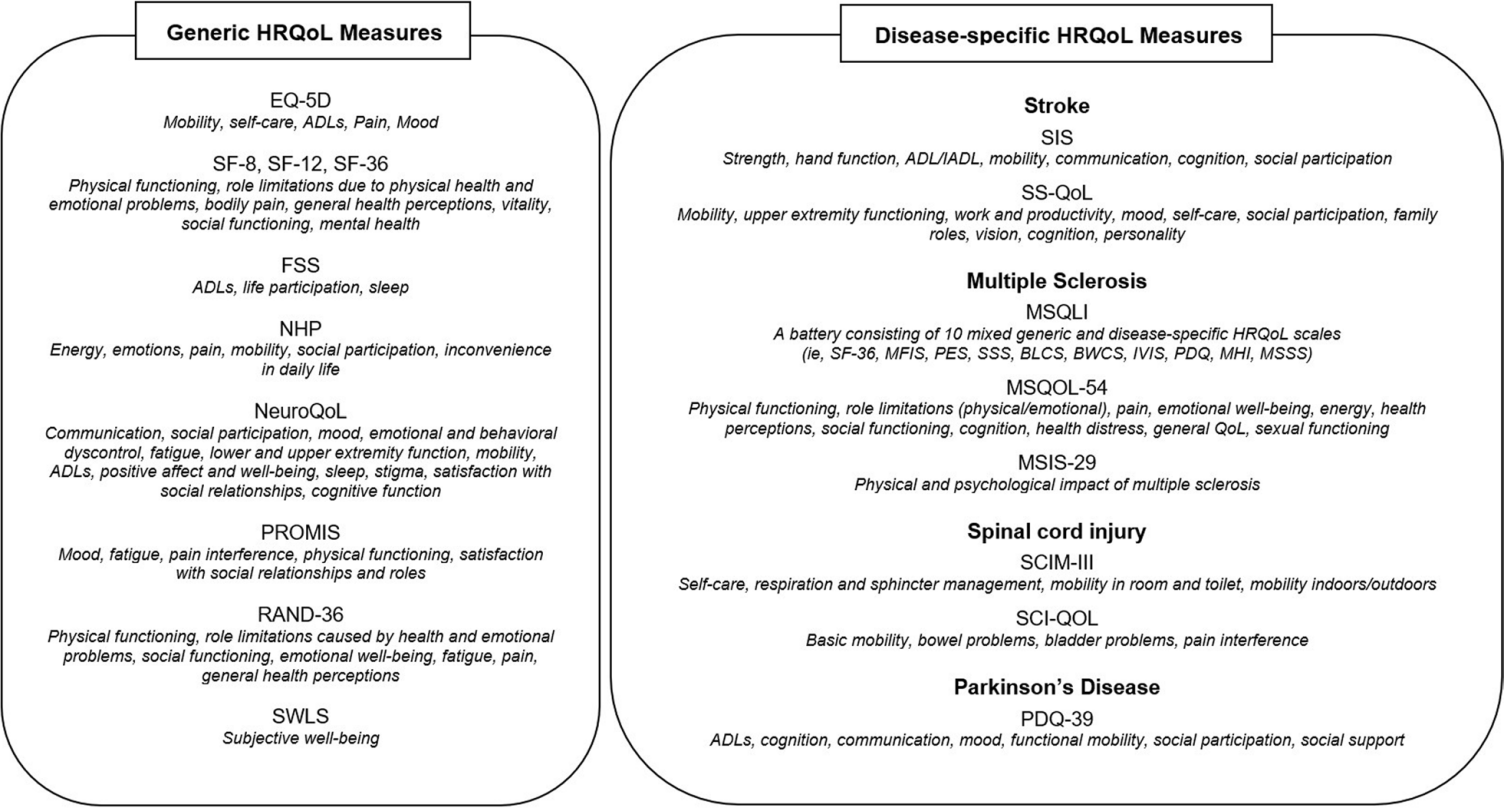
Summary of the generic and disease-specific HRQoL measures and their subcomponents investigated in the studies included. *BLCS* Bladder Control Scale, *BWCS* Bowel Control Scale, *EQ-5D* EuroQoL-5 Dimensions, *FSS* Fatigue Severity Scale, *IVIS* Impact of Visual Impairment Scale, *MFIS* Modified Fatigue Impact Scale, *MHI* Mental Health Inventory, *MSIS-29* Multiple Sclerosis Impact Scale, *MSQLI* Multiple Sclerosis Quality Of Life Inventory, *MSQOL-54* Multiple Sclerosis Quality Of Life-54, *MSSS* Modified Social Support Survey, *Neuro-QoL* Quality of life in Neurological Conditions, *NHP* Nottingham Health Profile, *PDQ* Perceived Deficits Questionnaire, *PDQ-39* Parkinson’s Disease questionnaire-39, *PES* Pain Effects Scale, *PROMIS* The Patient-Reported Outcomes Measurement Information Systems, *RAND-36* Research and Development Corporation-36, *SCI-QOL* Spinal Cord Injury-Quality of Life Independence, *SCIM-III* Spinal Cord Independence Measure-Version 3, *SF-8* SF-12, SF-36, Short Form Health Survey, *SIS* Stroke Impact Scale, *SS-QOL* Stroke Specific Quality Of Life Scale, *SSS* Sexual Satisfaction Scale, *SWLS* Satisfaction With Life Scale

A summary of the HRQoL measures used in the included studies is reported in Fig. 2. Notably, both generic and disease-specific HRQoL measures were administered. Most of the studies (55.7%) implemented disease-specific scales. Only three studies [81, 87, 89] provided a mixed evaluation. Different factors explaining HRQoL were investigated. Overall, these were associated with different health-related domains, including perceived physical functioning, emotional state, self-care, mood, pain, social participation, autonomy in the ADLs, and cognition.

### HRQoL changes in RAT

More than half of the studies (63.4%) reported significant effects on HRQoL following RAT alone. Of these, significant post-intervention between-groups changes were mostly found in stroke patients [32–34, 44, 46, 53, 65, 93], and in only one study in patients with PD [59]. Exclusive significant within-groups effects were found across clinical populations, namely stroke [42, 43, 45, 47, 54, 56, 58, 60, 84, 86, 87], MS [67, 72, 76, 82, 88, 89], SCI [30, 31, 39, 73], PD [68], and other neurological diseases [27, 63]. The 51.9% of the studies included follow-up evaluations. Of these, only two studies [44, 53] (both on patients with stroke) found significant between-groups effects over time (up to 3 months), while other studies on patients with stroke [47, 56, 60, 84, 86, 87] or MS [67, 89] showed only significant within-groups long-term effects (up to 36 months). Notably, most of the studies that reported significant effects implemented disease-specific HRQoL measures (60.6%). Similarly, 68.4% of the studies reporting no significant changes used generic HRQoL scales.

### HRQoL changes in RAT plus VR

The majority (66.7%) of the studies combining RAT with VR reported significant post-treatment changes on HRQoL. Significant between-groups effects emerged in patients with stroke only [13, 70, 91], while exclusive significant within-groups changes were observed in patients with stroke [49, 57, 66, 69, 79, 83] and MS [61, 64, 85]. No studies on the effects of RAT plus VR in patients with PD were retrieved. Moreover, only 33.3% of the studies on RAT plus VR included longitudinal evaluations. Of these, one study on patients with stroke [70] found significant between-groups effects over one month after intervention, while other studies on patients with stroke [57, 66, 69, 79] or MS [64] showed significant within-groups improvements up to 2 months after treatment. Similarly to studies on RAT alone, most (83.3%) of the significant effects were found when disease-specific HRQoL measurements were adopted.

### Non-motor outcomes concurrently investigated

Of the included studies, 32.9% conducted a psychological, cognitive and/or formative evaluation. Five studies investigated anxiety and depression symptoms [27, 63, 67, 68, 74], while ten assessed depression symptomatology only [13, 33, 41, 50, 54, 55, 58, 81, 82]. Seven studies conducted a cognitive assessment, especially concerning memory, attention, and executive functions abilities [13, 33, 34, 64, 68, 77, 83]. Other studies investigated patient satisfaction with the treatment [73, 77], perceived technological device usability [75], fear of falling [51], motivation and self-efficacy [71], coping abilities [33], and perceived well-being [33, 88].

## Discussion

A systematic review of the studies investigating the effects of RAT alone and RAT plus VR on HRQoL in patients with neurological diseases was conducted. From each study, a wide range of data were extracted, including the methodology adopted to measure HRQoL across neurological populations and a summary of the psychological outcomes concurrently evaluated. At the end of the review process, a total of 70 studies were included and synthetized. Of these, 52 studies were on RAT exclusively, while the remaining 18 examined HRQoL changes after implementing VR systems, too.

Over the last two decades, the number of studies including HRQoL as a rehabilitation outcome has been increasing sensibly. Indeed, most of the included studies date back to within the last five years, denoting a rapid and recent growing interest in investigating HRQoL and its subcomponents along motor outcomes. This reflected the composition of the research groups and their specialty field. Although it was observed that the majority had expertise in rehabilitation medicine and neurology, authors’ contributions came from different fields, such as engineering, neuroscience, occupational physiatry, physiology, geriatrics, public health, and psychology. The increase found in the number of studies and the heterogeneity of expertise area may be attributable to the increasing necessity and interest in adopting multidisciplinary approaches to study neurorehabilitation processes, especially when multiple outcomes that are not strictly related to motor improvement are considered and technological and innovative procedures are implemented [20]. Besides, the introduction of high technologies has however raised cost-effectiveness issues, particularly when referring to robotic devices. Prior evidence regarding the economic evaluations of technology-based rehabilitation interventions has so far drawn controversial conclusions within different neurological populations [94, 95]. Certainly, the implementation of robotics in rehabilitation programs entails higher costs when compared to more standard and conventional treatments, leading research on this topic to require financial support, especially when the feasibility of the technological devices is tested. This may explain why most of the included studies were funded and one study out of three was a pilot. In support of this, it was also observed that most of the studies implementing also VR received funding and provided preliminary evidence of technology deployment.

Further heterogeneity was also found in terms of patients’ characteristics, intervention, and robotic typologies used. Most of the patients had stroke, while others presented MS or SCI. Stroke prevalence is not surprising considering the nowadays global estimation of cases (70% over 64 years) with an increasing burden in both sexes [96, 97]. Regarding MS and SCI, it is widely recognized that both pathologies result in irreversible motor dysfunction often highly correlated to progressive disability and gait impairment [98, 99]. Accordingly, the number of cases observed in this systematic review reflects the clinical potential of RAT that has been so far exploited to target lower limb deficits (e.g., robot-assisted gait training—RAGT).

Furthermore, sample size varied extensively across the studies included. Although a considerable number of patients was included in this systematic review, more than one study out of three involved fewer than 30 participants leading to statistically underpowered results in most cases. Even though it must be noted that most of these studies were pilots, future research involving larger sample size to obtain more robust results is strongly recommended.

Similarly, it should be also highlighted that only four studies have reported patients’ ethnicity. As recommended by prior studies, future research is encouraged to report participants’ ethnic diversity for different reasons, including the possibility to provide increased results generalizability [100]. Lastly, informative findings regarding the intervention and the technology type implemented were observed. Overall, the studies varied considerably in the global intervention duration, total number of therapy sessions and individual session duration. Notably, differences in intervention procedures between RAT and RAT plus VR were found. On average, the rehabilitation programs coupling RAT to VR were shorter, suggesting that the concurrent implementation of VR systems may add complexity to recovery procedures, ultimately raising feasibility and usability issues [101]. In support of this, it was observed that all the studies on RAT plus VR used non-immersive VEs (e.g., PC monitors). It is acknowledged that the implementation of VR devices providing more immersive VEs was associated with a stronger likelihood for the patient to experience adverse effects like nausea, dizziness, or oculomotor disturbances, that in turn may represent factors potentially affecting RAT delivery and efficacy, as well as patients’ treatment acceptability. Nevertheless, although immersion is not strictly related to sense of presence and stronger engagement, it is recognized that it can lead patients to experience positive emotions and increased motivation during the treatment [102]. This would contribute to enhancing patients’ adherence to the therapy, ultimately optimizing its efficacy. Accordingly, future studies should more deeply explore the role of VR and its key characteristics (i.e., interactivity, immersion, and sense of presence) when combined with robotic devices and their effects on diverse non-motor outcomes, including HRQoL.

Mixed results on HRQoL were found. Regardless of the study design adopted, most of the studies reported significant effects following RAT alone or RAT plus VR. In both cases, the majority provided significant within-group improvements, and a lower percentage of studies reported significant post-treatment comparisons with a control group. Informative findings were then observed when examining HRQoL results with reference to the neurological population investigated, assessment methodology adopted, and study design. Although most of the studies provided significant evidence, both intervention typologies provided between-group effects in patients with stroke mainly. Evidence on the other pathologies (i.e., MS, SCI, and PD) were found with no significant differences to a control group. Moreover, of the studies coupling RAT to VR, only one involved patients affected by SCI and none on patients with PD were retrieved. Therefore, not only more studies involving larger sample sizes that allow study group comparisons are needed, but future research on this topic should also extend the investigation to neurological populations that have been understudied so far. Deeper observations of RAT effects on HRQoL are also needed to point out possible explanations about non-significant results. It must be noted that one study out of three found no significant effects in any neurological population. One possible explanation may be related to the methodology adopted to assess HRQoL. A large number of tools to assess a wide range of health-related components was retrieved and grouped into two main categories, namely generic and disease-specific HRQoL measures. The comparison between these two categories has been extensively discussed [103]. Although generic measures are designed to be broadly applied across different types and severity of diseases, they may generate contradictory evidence in content validity resulting in lower responsiveness in certain clinical populations. Disease-specific measures, on the other hand, might be more sensitive and representative when investigating HRQoL outcomes in specific clinical subgroups. In the present systematic review, it was observed that most of the studies that provided non-significant results adopted generic tools, whereas most of the significant effects were detected when disease-specific scales were implemented. Future studies should take this finding into account and, for example, adopt both measure types when assessing HRQoL to point out any internal consistency issues and advance more generalizable conclusions. Lastly, concerning the study design, it was noted that the studies reporting significant changes at follow-up evaluations (up to 36 months after treatment) were mainly on patients with stroke and, again, adopted a disease-specific HRQoL assessment approach. This supports the importance of the choice of HRQoL measures when targeting effects over time and, moreover, the necessity to extend longitudinal investigations to other neurological populations.

One last finding of this systematic review regards the psychological and cognitive outcomes investigated along HRQoL. Of these, only which variables and the related adopted measures were presented to give more centrality to the results on HRQoL. From data synthesis, it emerged that psychological outcomes such as anxiety and depression symptoms, coping abilities, motivation, or perceived device usability were mainly investigated in studies on RAT alone. Interestingly, patients’ cognitive aspects were mainly evaluated when VR was also applied to the treatment. This distribution is not surprising if we consider the impact that the exposure to VEs may have particularly on patients’ cognitive status given the potential of VR to provide multimodal stimulation and multisensory feedbacks during therapy. Nevertheless, future research should consider comprehensive evaluations of patients’ health domains in order to better understand their inter-relationships regardless of the type of intervention. Furthermore, future works are needed to better deepen also the relationship between the psychological and cognitive variables and specific HRQoL subcomponents.

### Limitations and future steps

Overall, the studies included in this systematic review made it possible to describe informative and promising evidence on robotic and VR devices application to neurorehabilitation and their effects on patients’ HRQoL. However, some limitations were identified too. The heterogeneity observed among the studies concerning the design, participants’ characteristics, intervention (i.e., rehabilitation outcomes, robotic device typology, duration), and HRQoL measures adopted made it especially difficult to draw robust conclusions. Moreover, this heterogeneity prevented advancing definitive interpretations on the added value of integrating VR to RAT. More studies combining the two technological devices are needed in order to provide more generalisable insights. Differently, the present review shed light on understudied neurological populations and, moreover, it allowed to note that, in the field of neurorehabilitation, HRQoL was investigated taking into account multiple and different health domains, ranging from motor and functional status to psychosocial related factors. The complexity of HRQoL as a construct, along with the other characteristics of the included studies, made the overall studies configuration too mixed to identify subgroups or subsets and, therefore, to perform meta-analyses as well. Accordingly, future research aiming at studying HRQoL improvements should take this heterogeneity into account and provide deeper investigations on specific HRQoL subcomponents and neurological populations adopting precise intervention procedures and an adequate assessment methodology. Following this line, multidisciplinary approaches are strongly recommended to optimally address the complexity of HRQoL and to extend knowledge on RAT effectiveness in patients’ everyday life.

### Supplementary Information

**Additional file 1: Appendix 1.** Summary of the data extraction and results of methodological quality assessment.

## Data Availability

Not applicable.

## Abbreviations

RAT: Robot-assisted therapy
VR: Virtual reality
HRQoL: Health-related quality of life
VE: Virtual environment
ADLs: Activities of daily living
PRISMA: Preferred reporting items for systematic reviews and meta-analyses
TBI: Traumatic brain injury
SCI: Spinal cord injury
MS: Multiple sclerosis
PD: Parkinson’s disease
